# Divorces in Connecticut

**Published:** 1888-08

**Authors:** 


					﻿EDITORIALS AND MISCELLANEOUS.
Divorces in Connecticut.—It appears from a recent report aggregating
the estimates of all the Town Clerks in the State of Connecticut that the per-
centage of divorces in that State is i to every 14.2 marriages!
				

